# Learning Curve and Comparison of Dynamic Implant Placement Accuracy Using a Navigation System in Young Professionals

**DOI:** 10.3390/dj10100187

**Published:** 2022-10-02

**Authors:** Johannes Spille, Eva Helmstetter, Paul Kübel, Jan-Tobias Weitkamp, Juliane Wagner, Henning Wieker, Hendrik Naujokat, Christian Flörke, Jörg Wiltfang, Aydin Gülses

**Affiliations:** Department of Oral and Maxillofacial Surgery, UKSH—Campus, Christian Albrechts University, 24105 Kiel, Germany

**Keywords:** navigation system, dynamic implant placement, learning curve, young professionals

## Abstract

The aim of the current study was to evaluate the learning curve and accuracy of implant placement by young professionals using a dynamic computer-assisted surgical system for dental implant placement. Ten students tried to place eight implants with a dynamic surgical system in predefined positions on two consecutive weekends, resulting in 160 implant placements in total. Postoperatively, the positions of the implants were scanned with an intraoral scanner and compared for deviations at the entry point, the apex, as well as angular deviations to the master model. The mean values of all measurements improved; statistical significance was found for the changes in the angle as well as for the position of the implants to the apex (*p* < 0.001). Furthermore, the young professionals indicated subjective improvement in handling the dynamic surgery system. Navigated surgical dental implant placement can be learned quickly and can support young professionals in everyday clinical practice, especially in difficult anatomic situations.

## 1. Introduction

Prosthetic restoration after dental rehabilitation with implants for optimal aesthetic restoration has a special significance in dentistry and requires high surgical accuracy [1]. Conventional implant placement is performed freehand or using laboratory-made surgical guides. Both are established procedures in dental recovery. Clinically good results and patient satisfaction can be achieved in most patient cases with these procedures [2]. However, these surgical procedures have limitations and operative risks as well, especially in difficult anatomical situations such as the atrophic mandible [3]. Application errors, which are more common for younger surgeons, seem to be the main reason for malfunction [4]. Implant malposition leads to peri-implantitis and craniomandibular dysfunctions, as well as implant loss [5].

Over the last two decades, navigation systems for implantation have constantly evolved to solve these problems and are increasingly used in clinics. Initial weaknesses such as the size of the devices, complicated handling, and errors in the hardware and software significantly improved [6]. Nowadays, navigation systems enable a good alternative in dental surgery. The anatomical situation of patients’ implant region is matched with the digitized prosthetic planning system using three-dimensional imaging such as computed tomography [7]. Computed-navigation systems enable the planning of positions of implants before surgery and the adjustment of the positions in real time by visualizing the anatomic situation on a screen [8]. This technique can reduce the risk ofinjury to important anatomical structures, such as nerves, vessels, or soft tissue [9]. Compared to fully guided static navigation, surgeons can take intraoperative, situational changes into account [10]. Nevertheless, the new technique would need to be learned by experienced surgeons, which may be a reason to reject the application in daily clinical practice. However, for young professionals, dental implant surgery could become easier with the help of navigation systems [11]. Further development and adaptation of navigated implantation using virtual and augmented reality technologies should gain more impact in dental surgery [12]. Usually, 3D computed-tomography data and surgical templates taken before the implants are inserted greatly facilitate the implantation in the correct position. However, there are anatomical situations in which these established surgical procedures reach their limits, and sufficient surgical experience is required to assure optimal implant placement. Therefore, the navigation technique is a great method to promote inexperienced surgeons in their practical preparation. For this reason, younger dentists should learn to use navigation systems in their surgical training or during their studies to be able to offer the entire repertoire of dental implant surgery.

The current study aimed to assess the learning curve and accuracy of implant placement by young professionals using a real-time navigation system. In this way, new technology can be demonstrated in the education of young professionals. Besides this, the technology can also be used to expose practicing surgeons to the digital workflow of implant placement to provide clinical care to patients with difficult anatomical conditions. The hypothesis of this study is that dynamic navigation allows for a more appropriate positioning of dental implants and shortens the learning curve even by unexperienced dental professionals.

## 2. Materials and Methods

### 2.1. Study Design

**Figure 1:**
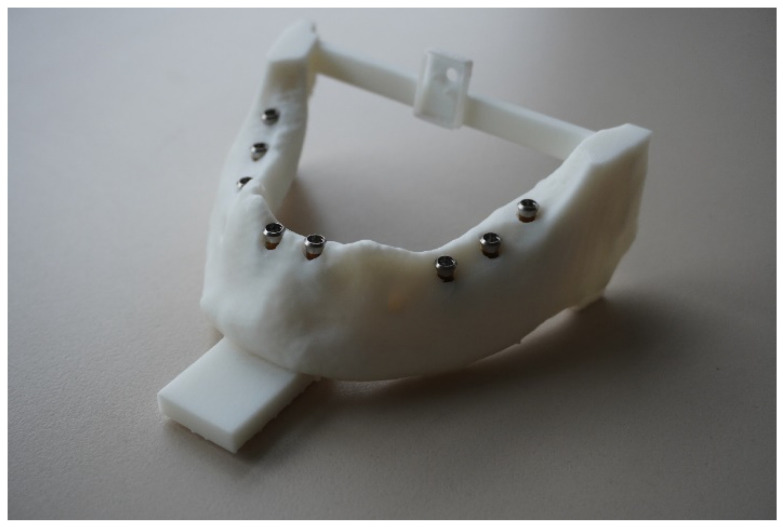
Imaged is the master typodont with the eight implants, which the young professionals had to implant in the same way with the dynamic computer-assisted surgical system. The prefabricated marker has to be placed in the acrylic structure in the middle of both rami of the mandible.

First, a toothless mandible was selected from the anatomical institute of the Christian-Albrechts-University in Kiel. The mandible was transferred into acrylic and duplicated several times. This allowed the same anatomical conditions for each participant to ensure reproducibility and optimal objectivity. Eight implants were placed in a master typodont (Figure 1). A cone beam computed tomography (CBCT) recorded the exact radiological topography of the inserted implants (KaVo 3D eXam, resolution: 0.2 voxels). This topography was transferred into the computer program coDiagnostiX (Dental wings GmbH, Chemnitz, Germany) and read into the Denacam navigation system of the company mininavident AG (Liestal, Schweiz, Switzerland).

Denacam is a dynamic computer-assisted surgical system and uses the principles of stereo triangulation of optical cameras. As a real-time navigation system, Denacam uses a small, prefabricated intraoral marker to coordinate the planned implant position and the real-time position of the drill during surgery. The prefabricated marker was placed between both rami of the mandible in acrylic. The surgeon detects deviations in the entry point, apex, and angle on a screen during surgery. Thus, the current drill position and the planned implant placement can be coordinated [11].

Ethical approval was obtained from the Ethic Commission of the Faculty of Medicine at the Christian-Albrechts-University, Kiel (D510/22).

### 2.2. Surgical Protocol

**Figure 2:**
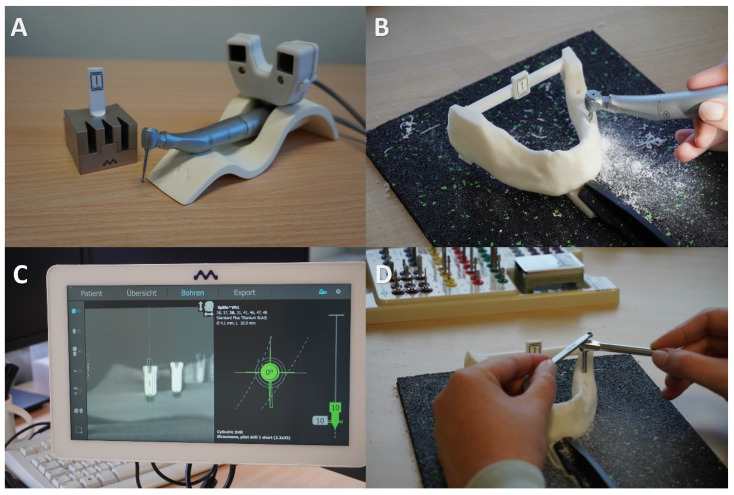
Illustrated is the dynamic workflow. In (**A**), a young professional registered the right drill, then the drilling could be performed with the Denacam navigation system (**B**). In (**C**), the young professional could see the right position of the drill in all relevant dimensions (angle, basis, apex). In (**D**), the young professional placed the implant in the drilled position.

The acrylic jaw was attached to the operating table. All students received individual instructions on implant placement and the function of the Denacam navigation system. The students followed the standard Straumann drill protocol during the surgical procedure; a 1.4 mm round burr was set to define the entry point first. A 2.2 mm pilot drill followed by a 2.8 mm pilot drill, a tap drill and 3.5 mm twist drill were used. Eight Straumann^®^ Standard Plus Tissue Level implants with a length of 10 mm and a diameter of 4.1 mm were placed in each acrylic jaw (Straumann Holding AG, Base, Switzerland) (Figure 2).

None of the students had experience in implant placement and performed their first implant operation on the acrylic jaw. After the first surgery, the students had the opportunity to practice with the Denacam navigation system. One week later, the students were able to practice again prior the second operation. Once the students felt ready, the second surgery was performed.

### 2.3. Evaluation of the Implant Accuracy

**Figure 3:**
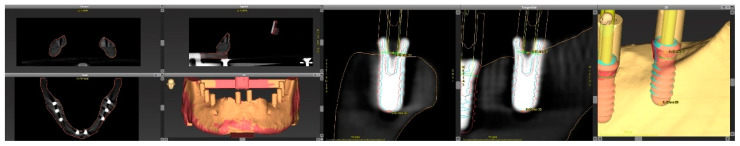
On the left side is a scanned and matched acrylic mandible of a student with inserted implants and screwed SmartPegs. On the right side are the computed and protocolled values.

Each operated acrylic jaw was compared with the master typodont. A SmartPeg Type 04 from the company Osstell (Gothenburg, Sweden) was placed on each implant and scanned with the intraoral scanner TRIOS^®^ from the company 3Shape (Copenhagen, Denmark). The data were evaluated and calculated with the coDiagnostix software afterward (Figure 3):

- Total error at the basis/entry point (vestibular, lingual, mesial, distal deviation);
- Total error at the apex (cranial, caudal deviation);
- Angular error.

The values of the individual implants from the first and last acrylic jaw each student worked on were compared. The evaluation was used to describe the learning curve of the students.

Furthermore, a short questionnaire was evaluated regarding the subjective reliability of implant placement. The questionnaire was completed by all students after the first and second day of surgery. The students described in the questionnaire what problems they encountered during implant placement in both sessions, so the individual and subjective success of each student in the second session could be identified. In this regard, the learning curve could independently checked in accordance with the results and reasons for a change could be given.

### 2.4. Statistical Analysis

Statistical analyses were performed using SPSS (IBM^®^, Ehningen, Germany). Normally distributed and non-normally distributed continuous variables were expressed as mean (±SD), and categorical data were presented as total counts. The ratio of deviations in the entry point, apex, and angle was calculated. The relation between variables was evaluated by sample *t*-test. Associations were considered significant when the *p*-value was <0.05.

## 3. Results

**Figure 4:**
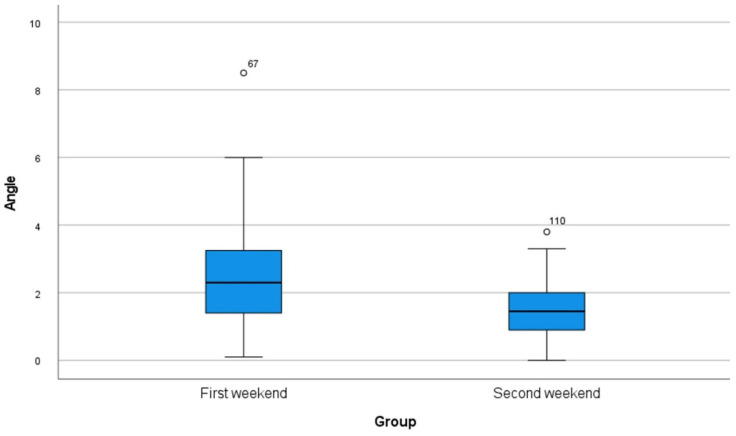
Box plot of all measurements of deviations in the angle regarding the master typodont.

**Figure 5:**
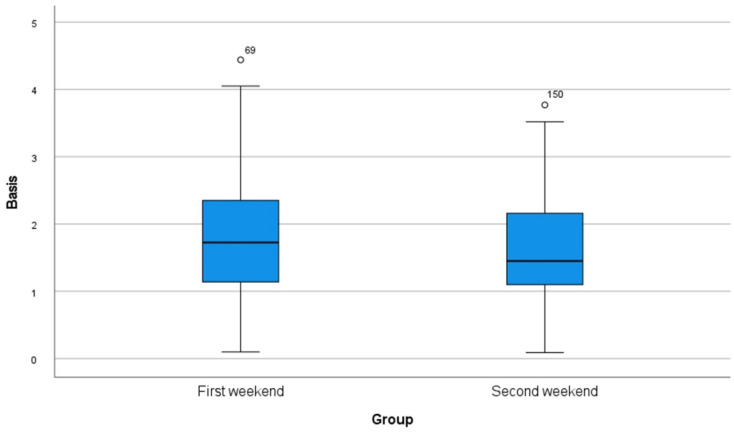
Box plot of all measurements of deviations in the exact positions in vestibular/oral and mesial/distal direction (basis) regarding the master typodont.

**Figure 6:**
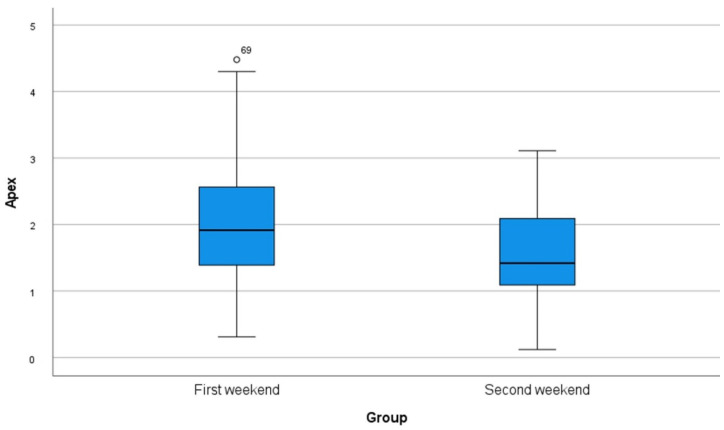
Box plot of all measurements of deviations in the exact positions in cranial/caudal direction (apex) regarding the master typodont.

A total of 80 implants were inserted on each of the weekends (10 students, implants *n* = 8). The mean values and standard deviations of each parameter are shown in Table 1 as well as in Figure 4, Figure 5 and Figure 6. Statistical significance was found for the changes in the angle as well as for the position of the implants to the apex (*p* < 0.001). Although no significance was found for the position of the implants to the basis (*p* = 0.161), there was a clinical improvement.

**Figure 7:**
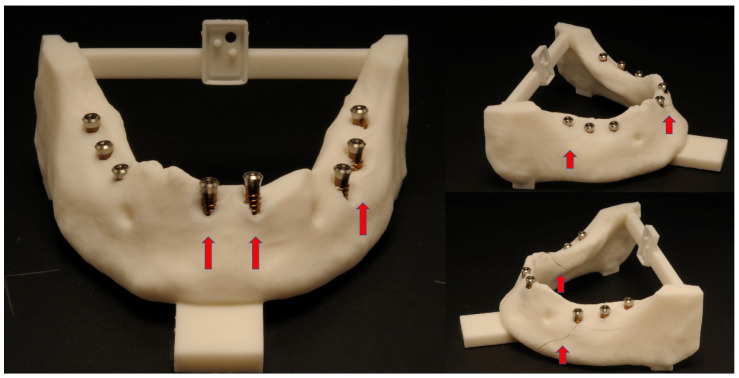
The main problems during the first session were that the acrylic jaw fractured due to excessive pressure or mishandling of the implant placement. Furthermore, the correct depth was not always achieved with the drills. Some of these mistakes were shown with the red arrows.

**Figure 8:**
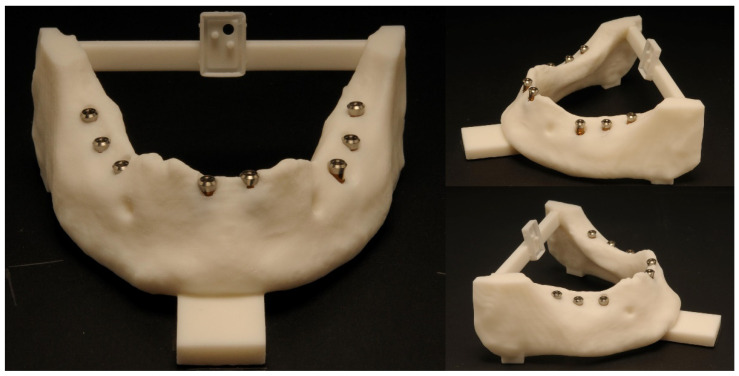
Illustrated are acrylic mandibles of three different young professionals on the second session. Considerably less problems were seen on the second session; most implants could be inserted in a satisfying position.

Table 2 and Table 3 show the responses from the questionnaire of the ten students and their subjective perception of the improvement in handling the mininavident system on the second weekend. Furthermore, the students declared their difficulties in a free response section of the questionnaire. There were problems with the coordination of the handpiece and the monitor and the detection of the marker with the camera. Other problems were the weight of the handpiece and the field of view in the right distal mandible. On the second weekend, the students declared fewer problems with the operation procedure. Figure 7 and Figure 8 show some of the postoperative acrylic mandibles and present the problems, as well as the successes and the steep learning curve of the students, respectively.

## 4. Discussion

This study demonstrated substantial improvement in dental implant placement with the dynamic computer-assisted surgical system, operated by young professionals. For the students, this was the first implant placement, and the toothless acrylic jaw presented a difficult anatomical situation because no teeth or prosthetic restorations were available for orientation. Significant values were shown for the position at the apex and the angular position as well as there was an increasement for the position at the basis. Golop Deep et al. described similar results; young professionals improved significantly in speed and angulation deviation by using a dynamic navigation system [13]. Sun et al. described a steep learning curve for longitudinal and angular deviations for an experienced dentist [14]. Marques-Guasch et al. saw a decreased learning curve for a young inexperienced surgeon and concluded that the navigation technique requires a lot of practice to learn the right hand–eye coordination [15]. However, dynamic navigation could be a good alternative to the standard procedures and could enable young dentists to perform targeted and reliable therapy that is gentle on the patient [16].

Initially, the dynamic systems were used only with remaining teeth, but Feng et al. used temporary mini-implants to affix the required marker needed to place dental implants in the interforaminal region. After repeated operations with the dynamic system by an experienced surgeon, the accuracy of the implant positions improved significantly [17]. To the best knowledge of the authors, this study was the first to evaluate the accuracy and learning curve of dental implant placement by young professionals with a dynamic computer-assisted surgical system in a toothless acrylic jaw, copied from an elderly patient.

The dynamic navigation system enabled the students to place dental implants in good positions with little practice. Usually, the accuracy of virtually planned and conventionally placed implants depends on the experience of the surgeon [18]. Certainly, this study is only performed on acrylic mandibles and the handling of the system was performed outside the mouth, but experienced surgeons also demonstrated a learning curve for dynamic dental placement to achieve satisfactory results in vivo [19]. Nevertheless, students seem to be able to achieve steeper learning successes, especially with dynamic navigation [20]. While this operation procedure seems easy to master, it must be kept in mind that deviations are greater in vivo due to disruptive factors such as limited mobility, restricted mouth opening, or patient movement [21].

In the current study, the young professionals felt much more confident with the implant placement as well as the handling of the navigation system after the first weekend and another practice time on the second weekend. Only the field of view during operation appears to remain a challenge. The positioning of anterior implants seems to be easier to learn. As a right-handed person, the view of the left side is restricted (especially right posterior mandible), which makes exact drilling more difficult [13].

Furthermore, the young professionals described the coordination of the handpiece with the monitor as a problem. This could be due to the weight of the handpiece, which is occupied by the camera and complicates the operation. In addition, the detection of the marker with the camera was not easy to perform. The marker as an additional intraoral medium can also negatively influence the view. Observing the patient and the monitor correctly during the entire operation can lead to complications and inaccuracies [22,23].

On the first weekend, the students had problems with the acrylic mandible chipping and fracturing. The surface as well as the bone quality are of high importance for the accuracy of implant placement. Surgeons must learn the different patient conditions during education because every patient’s anatomy is different. Wittwer et al. described that an irregular bone condition may lead to uncontrolled shifting of the drills during surgery [24]. In addition, the type of insertion can determine the temperature formed in the bone. Gurdán et al. described that higher temperature values during implantation reduced the survival of dental implants and led to thermal osteonecrosis [25]. Finally, further surgical circumstances and conditions determine the success of dental implantation, such as the contact pressure, the wear and type of the drill or the drill speed [26].

Ultimately, a higher level of surgical experience with dynamic navigation improves the accuracy of implant placement and may also provide a good alternative in daily surgical treatment; the dynamic computer-assisted surgical system enables dentists to plan and operate on the patient quickly [27]. However, most surgeons still use conventional surgical templates, considering the cost and complexity of computer-aided guidance [28]. In many cases, this technique is adequate, but in some cases, it can have limitations [29]. Conventional surgical templates do not offer a solution for difficult anatomical conditions and insufficient field of view, so the surgeon must resort to the freehand technique [30]. Some studies have shown that the accuracy of dental implant placement with surgical navigation systems is superior to that of freehand insertion [28,31]. Zhan et al. concluded that the learning curve was steeper with the dynamic navigation technique than with the freehand technique [32]. Anatomical structures that deviate from the norm can be reasons for major inaccuracies during freehand implant placement [33] and the rate of complications could be reduced by highly accurate implant placement with dynamic surgery systems [34]. This is the reason why especially young professionals could benefit from this new technique and should learn the dynamic dental surgery in their residency. Finally, Hassfeld and Mühling recognized already in 2001 that computer technology could provide precise and reliable support to oral surgeons, as it enables much better orientation and handling, especially in difficult anatomical situations [35]. Dynamic navigation requires a higher radiation dose, more preoperative planning and higher costs; however, dynamic navigation already shows good in vivo results and offers an alternative for implant placement in complex patient cases, as well as for prosthetic restoration after extensive tumor surgery [27,36,37]. Thus, it is the responsibility of universities to show students the newest technologies and provide training for them.

The current study had also some major limitations. First, because the study was based on a small group of ten young professionals, a larger cohort study is needed to improve statistical power. Besides that, not all anatomical structures such as the inferior alveolar nerve or prosthetic sense were considered in the implant position. Furthermore, the lab conditions cannot be accurately compared to real life. The learning curve in real life happens on different patients with different anatomy and bone quality. Thus, also several types of jaw typodonts with wider problems should be compared. In the future, a large database or a prospective in vivo study for dynamic dental implant placement should be established.

## 5. Conclusions

The current study showed that a dynamic computer-assisted surgical system can be a good alternative in dental implant placement, especially in difficult anatomic situations. Navigated surgery in dental implant placement has a steep learning curve. Young professionals could be acclimated to this novel technology from the very beginning of their training and could use it for difficult operations in the interest of patient well-being. Finally, further studies are needed to ensure a significant conclusion about the advantages of the method for young professionals. However, training in navigated dental implantation should be encouraged and could be a helpful part in residency.

## Figures and Tables

**Table 1:** Imaged are the mean values and deviations of all students from the master typodont. The deviations of the angles of the implant positions and the exact positions in vestibular/oral and mesial/distal direction (basis) as well as in cranial/caudal direction (apex) were calculated.

	Total Error at Basis (Mean ± SD)	Total Error at Apex (Mean ± SD)	Angular Error (Mean ± SD)
First session	1.80 mm ± 0.93 mm	2.02 mm ± 0.88 mm	2.51° ± 1.48°
Second session	1.61 mm ± 0.81 mm	1.56 mm ± 0.70 mm	1.51° ± 0.82°

**Table 2:** Responses of the ten students regarding subjective reliability in using a dynamic computer-assisted surgical system in dental implant placement (first session).

	Very Safe	Safe	Sufficient	Unsafe	Very Unsafe
First session How safe did you feel during implant placement?		3	4	2	1
How safe did you feel with the handling of the mininavident?	3	4	2	1	
How safe did you feel with the field of view during operation?		2	1	5	2

**Table 3:** Responses of the ten students regarding subjective reliability in using a dynamic computer-assisted surgical system in dental implant placement (second session).

	Very Safe	Safe	Sufficient	Unsafe	Very Unsafe
First session How safe did you feel during implant placement?	2	3	5		
How safe did you feel with the handling of the mininavident?	3	6	1		
How safe did you feel with the field of view during operation?		4	3	3	

## Data Availability

The data presented in this study are available on request from the corresponding author.
